# The risk of stroke according to statin medication compliance in older people with chronic periodontitis: an analysis using the Korea National Health Insurance Service-Senior Cohort Database

**DOI:** 10.4178/epih.e2022055

**Published:** 2022-07-05

**Authors:** Seon-Rye Kim, Minkook Son, Yu-Rin Kim

**Affiliations:** 1 Department of Healthcare Management, Youngsan University, Yangsan, Korea; 2 Department of Physiology, College of Medicine, Dong-A University, Busan, Korea; 3 Department of Dental Hygiene, Silla University, Busan, Korea

**Keywords:** Ischemic stroke, Periodontitis, Hydroxymethylglutaryl-CoA reductase inhibitors, Compliance, The aged

## Abstract

**OBJECTIVES:**

We investigated the risk of stroke according to statin medication compliance in older people with chronic periodontitis.

**METHODS:**

Chronic periodontitis patients were extracted from the National Health Insurance Service-Senior Cohort Database from 2002 to 2014. Among 255,056 chronic periodontitis patients, 41,412 patients with statin prescriptions for 28 days or more were included. The study population was divided into the top 25% of medication compliance group (TSG) and the lower 25% of medication compliance group (BSG). After 1:1 propensity score matching was performed, the final number of patients in the BSG and TSG was 6,172 each. To analyze the risk of stroke, a Cox proportional hazard model was performed to calculate hazard ratios (HRs) and 95% confidence intervals (95% CIs) after adjusting for age, sex, income level, hypertension, diabetes, and Charlson comorbidity index.

**RESULTS:**

In the Kaplan-Meier curve, the disease-free probability was prominently lower in the BSG than in the TSG (p for log-rank= 0.001). The HR in the multivariable-adjusted model for stroke occurrence in the TSG compared to the BSG was 0.79 (95% CI, 0.67 to 0.92; p=0.002). Subgroup analyses showed significant associations between compliance to statin medication and stroke, especially in female, people 85 years or older, and patients with comorbidities.

**CONCLUSIONS:**

Increasing compliance to statins may reduce stroke risk in older adults with chronic periodontitis. Therefore, in order to increase medication compliance among older people with chronic periodontitis, it is necessary for medical staff to make efforts to provide effective medication guidance.

## GRAPHICAL ABSTRACT

[Fig f3-epih-44-e2022055]

## INTRODUCTION

Periodontal disease causes inflammation in the tissues around the teeth, forming periodontal pockets and ultimately causing tooth loss by resorption of the alveolar bone [1]. Periodontal disease is one of the most common medical conditions, with a reported global prevalence of 20-50% [2]. Periodontal disease begins as gingivitis, the risk of which gradually increases in adolescence and into adulthood. According to the 2019 Health Insurance Statistical Yearbook of the National Health Insurance Service (NHIS), gingivitis and periodontal disease ranked first in the incidence rate, and the prevalence of periodontal disease among adults decreased to 22.7% in 2012 from 35.5% in 2007. After this period of decline, it increased to 30.5% again in 2016-2018 [3]. The nonsurgical treatment of periodontal disease reduces periodontal pockets and increases the clinical adhesion level to some extent, but does not compensate for the loss of alveolar bone [4-6]. Therefore, periodontal regeneration treatment, which includes statin use, is being performed as an adjuvant therapy to reduce tissue destruction.

Statins, also known as β-hydroxy β-methylglutaryl-CoA reductase inhibitors, refer to a group of anti-hyperlipidemic drugs whose names end in -*statin*. Statins are widely used drugs for dyslipidemia and hyperlipidemia, and they are known to significantly reduce cardiovascular abnormalities and mortality caused by these diseases [7]. These effects have been demonstrated not only in patients with cardiovascular disease, but also in the prophylactic treatment of patients in risk groups without cardiovascular disease. Furthermore, statins combat periodontal disease and help control periodontal lesions in several ways, including anti-inflammatory effects [8,9], antibacterial activity [10], skeletal formation promotion and bone loss inhibition [11,12], and antioxidant properties [13]. Statins have also been used to treat stroke, and the American Heart Association and the American Stroke Association published guidelines for the secondary prevention of cerebral infarction to reduce the risk of recurrence in patients with cerebral infarction or transient ischemic attacks (TIAs). These guidelines emphasized that lipid therapy through statins should be applied [14,15].

Stroke is a collective term for local neurological deficits that are suddenly caused by abnormalities in cerebral blood flow [14]. As such, intensive lipid control therapy using statins was recommended to reduce the risk of stroke and cardiovascular events occurring in TIA patients, along with ischemic stroke of atherosclerotic origin (class I, level B) [15]. Although a correlation between cholesterol levels and ischemic stroke was confirmed in a previous cohort study, there was no clear association with total stroke [16]. The Plasma Lipid Profile and Incident Ischemic Stroke study [17] conducted 10-year prospective observations of more than 14,000 middle-aged people, but it only confirmed an inconsistent and weak correlation between cholesterol levels and ischemic cerebral infarction. In contrast, in the Multiple Risk Factor Intervention Trial study, which included 350,000 people, it was also reported that the risk of ischemic cerebral infarction increased as the cholesterol level increased [18]. Therefore, it is necessary to evaluate the effects of statins on reducing the risk of stroke in chronic periodontal disease patients.

When considering the effect of a particular treatment, it is often thought the result depends on whether the patient has properly taken the relevant medication. The full therapeutic effect of a drug can be achieved when the patient takes the drug according to the doctor’s prescription—that is, when the medication compliance is high [19].

Medication compliance is generally defined as “the extent to which a patient is taking medications as prescribed by their healthcare provider” [19]. Poor medication compliance can lead to aggravation of the disease and increases in additional medical utilization and expenses. These results are particularly prominent in chronic diseases, for which long-term drug use is the mainstay of treatment [20,21]. Despite the importance of medication compliance, the level of medication compliance among chronically ill patients in Korea is very low. For example, in the 2011 Korean Medical Panel, 68.0% to 86.9% of participants answered, “I tend to take the medication according to the prescribed method,” depending on the disease [22]. In addition, a 4-year follow-up study of patients with hypertension from 2003 to 2007 found that only 43.4% of patients had a medication possession ratio (MPR) of 80% or higher [23]. Moreover, a 2-year follow-up story of type 2 diabetes patients from 2004 to 2006 showed a very low medication compliance rate (29.4%) [24].

Medication compliance figured prominently in the present study due to its importance in treatment. We compared the risk of stroke between two groups: the top 25% of statin compliance group (TSG) and the bottom 25% of statin compliance group (BSG) among older people diagnosed with chronic periodontal disease. The information came from the Senior Cohort Database provided by the NHIS. Consequently, this study aimed to provide basic data on the risk of stroke according to statin medication compliance among older Korean patients with chronic periodontal disease.

## MATERIALS AND METHODS

### Subject selection

This study utilized the Senior Cohort Database provided by the NHIS. The Korea National Health Information Database (NHID) is a public database on healthcare services maintained by the NHIS of Korea, which is a universal health insurance system that covers the medical expenditures of approximately 98% of all Korean citizens [25]. The database includes representative and comprehensive information on medical use among Korean patients, including insurance eligibility, diagnostic codes, prescribed medications, procedures, and billing records [26]. From 2002 to 2014, among 255,056 chronic periodontal disease patients, 41,412 patients with statin prescriptions for 28 days or more were analyzed. With the exclusion of 5,288 patients with cerebrovascular disease and myocardial infarction, 99 patients without data, and 3,718 patients with chronic periodontal disease in 2002, 32,307 patients were extracted for analysis. In this study, the MPR was evaluated to distinguish individuals who took statins regularly from those who took statins irregularly.

**Figure 1.**
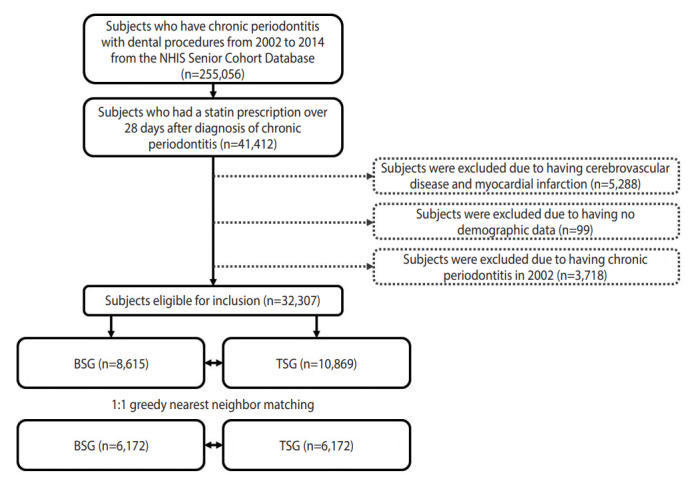
Flow chart of the study population. NHIS, National Health Insurance Service; BSG, bottom 25% of statin compliance group; TSG, top 25% of statin compliance group.

The MPR is calculated as the proportion of the number of days of treatment during the follow-up period from the first to last prescription. In this study, having the medication dispensed at the pharmacy was considered to indicate medication compliance. This measurement method has a limitation in that it is impossible to determine whether the patient actually took the drug or took it at the appropriate time. However, this method is known to provide a relatively accurate estimate of medication compliance in large patient groups using cohort data [19,27]. Therefore, the study population was divided into the top 25% of medication compliance (TSG; first quartile of MPR) and the lower 25% of medication compliance (BSG; fourth quartile of MPR). In order to ensure the homogeneity of the study subjects, 1:1 propensity score matching (PSM) was performed, and the final numbers of patients in the BSG and TSG were 6,172, respectively. The total number of study subjects was 12,344 (Figure 1).

### Definition of variables

The considered covariates were age, sex, income level, hypertension, diabetes, and Charlson comorbidity index (CCI). Chronic periodontitis was identified as an International Classification of Diseases, 10th revision (ICD-10) code of K053 [28] and the treatment codes for chronic periodontitis were defined as U2232, U2233, U2240, U1010, U4411, U4412, U1051, U1052, U1072, U1072, U1081, U1082, and U1083. Hypertension and diabetes were identified as covariates. Hypertension was defined as ICD-10 codes of I10 or I11 and diabetes was defined as ICD-10 codes of E10, E11, E12, E13, or E14. Hypertension and diabetes were also defined as being present in patients who were prescribed a corresponding medication at least once per year. Ischemic stroke was defined as a code of I63 or I64 with at least 1 hospitalization (Supplementary Material 1). The CCI was calculated based on underlying diseases, including myocardial infarction, congestive heart failure, peripheral vascular disease, cerebrovascular disease, dementia, chronic pulmonary disease, connective tissue disease, peptic ulcer, mild liver disease, diabetes with and without complications, paraplegia or hemiplegia, renal disease, any or metastatic cancer, moderate or severe liver disease, and acquired immune deficiency syndrome before the start of follow-up period [29,30].

### One-to-one propensity score matching

To ensure the homogeneity of the 2 groups, age, sex, income level, hypertension, diabetes, CCI, and disease to medication interval were applied as variables in 1:1 PSM. The PSM analysis was carried out on the sampled cohort with logistic regression to consider selection bias and the presence of potential confounding variables. The standardized difference in potential confounding variables was confirmed to be less than 0.025, indicating the homogeneity of the 2 groups (Supplementary Material 2).

### Statistical analysis

The Senior Cohort Database provided by the NHIS was analyzed using R version 3.6.0 (https://www.r-project.org/) and SAS version 9.4 (SAS Institute Inc., Cary, NC, USA). The baseline characteristics according to the TSG and BSG among patients with chronic periodontitis were compared using the Student t-test and the chi-square test. A Kaplan-Meier curve was presented for stroke risk analysis, and the log-rank test was performed. The incidence rate of ischemic stroke was presented in units of 1,000 person-years for the total follow-up period of the TSG and BSG. To analyze the risk of stroke, a Cox proportional-hazard model was performed to calculate the hazard ratio (HR) and 95% confidence intervals (CIs) after adjusting for age, sex, income level, hypertension, diabetes, and CCI. A p-value < 0.05 was considered to indicate statistical significance.

### Ethics statement

The study protocol was approved by the Institutional Review Board (IRB) of Silla University (IRB No. 1041449-202102-HR-001). The requirement for informed consent was waived by the IRB since personal information that can be used to identify individuals registered to NHID was removed.

## RESULTS

### Demographic characteristics according to medication compliance

The total number of study subjects was 12,344, and 687 stroke occurrences (5.6%) were confirmed during the median follow-up period of 5.3 years. No significant demographic characteristics between the TSG and BSG were found in age, sex, income level, hypertension, diabetes, and CCI (p>0.05). In addition, the interval from diagnosis of chronic periodontitis to taking statins was approximately 3 months, without a significant difference between the 2 groups (p=0.78). However, the MPR was significantly higher in the TSG than in the BSG (p<0.001). The stroke incidence was significantly lower in the TSG (4.5%) than in the BSG (6.6%) (p<0.001) (Table 1).

### Comparison of ischemic stroke risk according to medication compliance

**Figure 2.**
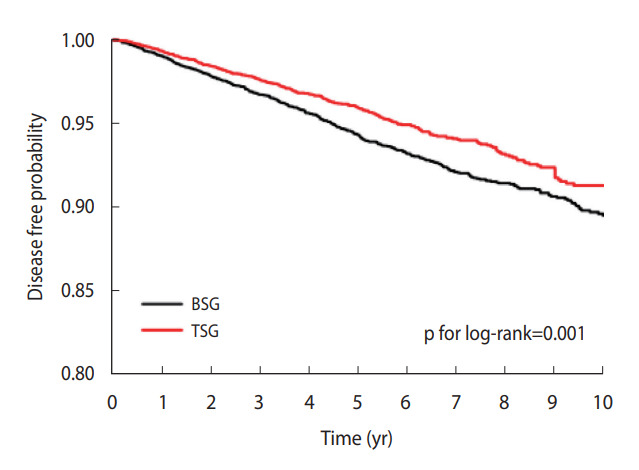
Kaplan-Meier curve for ischemic stroke and statin compliance. BSG, bottom 25% of statin compliance group; TSG, top 25% of statin compliance group.

In the Kaplan-Meier curve, the disease-free probability was prominently lower in the BSG than in the TSG (p=0.001 by the log-rank test) (Figure 2). Of the 12,344 study subjects, 408 stroke patients occurred in the BSG and 279 stroke patients occurred in the TSG. The incidence rate (per 1,000 person-years) was 11.47 in the BSG and 8.90 in the TSG. Stroke was significantly associated with statin medication compliance in the crude and multivariableadjusted Cox proportional hazard models (Table 2). The crude HR for stroke occurrence in the TSG compared to the BSG was 0.78 (95% CI, 0.67 to 0.91; p=0.001). The HR in the multivariable-adjusted model was 0.79 (95% CI, 0.67 to 0.92; p=0.002).

### Subgroup analysis according to sex, age, and comorbidities

Subgroup analyses were performed according to sex, age, and comorbidities including hypertension and diabetes (Table 2 and Supplementary Material 3). In the subgroup analysis by sex, the incidence rate of stroke in the BSG was higher than that in the TSG, regardless of sex. The adjusted HR for stroke in the TSG compared to the BSG was 0.87 (95% CI, 0.68 to 1.10) for males, but it was not statistically significant (p=0.24). In females, the adjusted HR for stroke was 0.73 (95% CI, 0.60 to 0.90), which was statistically significant (p = 0.002). In the subgroup analysis by age, with participants divided into early (65-74 years), middle (75-84 years), and late older age (≥ 85 years) groups, the incidence rate of stroke in the BSG was higher than that in the TSG, regardless of age. The adjusted HR for stroke in the TSG compared to the BSG was 0.78 (95% CI, 0.65 to 0.94) in the late older age group (p=0.01). The subgroup analysis by comorbidities showed significant associations between statin compliance and stroke in patients with hypertension or diabetes.

## DISCUSSION

In this large-scale and long-term follow-up cohort study, we confirmed the association between statin medication compliance and ischemic stroke in older people with chronic periodontitis. Previous studies reported that chronic periodontitis and stroke were related [31,32], but stroke may be affected by various factors. In particular, dyslipidemia had a clear influence on the occurrence of stroke to the extent that some of the guidelines for the primary prevention of stroke were revised [33]. Previous studies have simply confirmed the relationship between chronic periodontitis and stroke; however, few studies have analyzed the effects of dyslipidemia medications on stroke in patients with chronic periodontitis. Therefore, this study was conducted to confirm the effect of statins on the risk of stroke in patients with chronic periodontitis using the NHIS Senior Cohort Database, with the aim of providing information to support basic medical guidelines for statin use in patients with chronic periodontitis. The risk of stroke in older people with chronic periodontitis was higher in the BSG than in the TSG. These results are similar to those of Amarenco & Labreuche [34]’s meta-analysis of randomized clinical trials that compared a statin-use group and a control group (where no placebo nor statin was used) for stroke prevention. Their study reported an 18% reduction in stroke incidence in the statin-treated group, with no increase in hemorrhagic stroke. Their study is similar to ours in terms of demonstrating the effectiveness of statins, but a difference is that our study specifically analyzed statin medication compliance.

In Korea, which is aging rapidly, medication compliance among older people suffering from chronic diseases is a very important issue, both for preventing disease and improving treatment and for reducing the economic costs borne by the individual and national health system [35]. Medication compliance means taking medications on time according to the prescription [36]. Clearly, if medication is not taken properly, its usefulness as a pharmacological treatment is limited; therefore, medication compliance is crucial in the management of disease [37]. We found that the HR for ischemic stroke was higher in the BSG than in the TSG, even when adjusting for age, sex, income level, hypertension, diabetes, and CCI. Our results are similar to the study of Faught et al. [38], which investigated the risk of death according to medication compliance. They analyzed 33,658 patients registered with the Medicaid program in the United States who had been prescribed antiepileptic drugs more than twice in the past 8 years. Their results demonstrated the importance of medication compliance, as patients with a medication compliance rate of less than 80% for 3 months had an approximately 3 times higher risk of death than patients who regularly took their medication.

The subgroup analysis according to sex in this study showed that females had a lower incidence of stroke in the TSG than in the BSG, although without a statistically significant difference. However, females showed a statistically significantly lower incidence of stroke in the TSG than in the BSG. The clinical results after stroke treatment are generally known to be worse in females than in males [39]. Among patients who survive after stroke treatment, females are more likely to enter convalescent homes than male, and female tend to have more severe disabilities after treatment [40]. In the Justification for the Use of Statins in Prevention: An Intervention Trial Evaluating Rosuvastatin (JUPITER) study, the cardiovascular disease prevention effect of rosuvastatin was similar in male (95% CI, 0.45 to 0.73) and female (95% CI, 0.37 to 0.80) [41]. However, a meta-analysis of 13,154 female, including the JUPITER study, reported that statins reduced cardiovascular disease incidence in female by 37% (95% CI, 0.49 to 0.82) [42]. Based on these results, health care professionals may consider increasing statin medication compliance as a way to reduce the risk of stroke in female patients. In addition, when age was considered as a sub-item, this study found no statistically significant difference related to compliance with statins in the group under the age of 75. However, in the group over the age of 75 years, the incidence of stroke was significantly lower in the TSG than in the BSG. The Framingham Heart Study, which is the oldest among prospective cohort studies on cardiovascular disease and has contributed the most to the identification of stroke risk factors, revealed that the incidence of stroke increased by 200% for every 10 years of age after 55 years of age [43]. Therefore, when considering the results of this study, effective medication guidance should be provided to increase statin medication compliance as a way to reduce the risk of stroke in the older population. According to the Diabetes Fact Sheet in Korea 2020, older people often have comorbidities such as high blood pressure, dyslipidemia, and diabetes, and take an average of 8.2 drugs, which may increase their pill burden [44]. In the sub-item analysis of this study, the risk of stroke was lower in the TSG in the presence of comorbidities. The effects of statins on these comorbidities are consistent with existing studies [14,15,20,21,41,42]. As such, medication compliance with statins is very important as a way to reduce the risk of ischemic stroke in older patients with chronic periodontitis.

As with all research, it is valuable to examine the limitations and strengths of the present study. Despite having found results similar to other previous scholarly works, this study has some limitations to consider. First, the disease codes might not have accurately reflected patients’ medical conditions, as they are sometimes exaggerated to receive reimbursement due to a fee-for-service payment system [46]. Second, the risk of stroke was analyzed according to the medication compliance of statins, but the capacity or strength of statins themselves was not investigated. Lastly, limited covariates were applied for the risk of stroke. However, this study has some strengths. One advantage of this study is that it provides reliable data based on a large sample and a long follow-up period, thus making it representative of the entire older population. In addition, homogeneity between the 2 groups (TSG and BSG) was ensured through PSM.

In summary, there was a significant association between compliance with statins and stroke in older people with periodontitis. Accordingly, older people with chronic periodontitis should increase their medication compliance when taking statins in order to lower the risk of stroke. This is recommended especially for female patients and other older patients with a high risk for stroke. To best increase medication compliance among older people with chronic periodontitis, it is necessary for medical staff to make efforts to provide effective medication guidance. Additionally, caregivers who make home visits for older patients with reduced mobility will need to develop their capacity to provide effective medication guidance. Only with support from national health organizations and institutions will future efforts engender positive change.

## Figures and Tables

**Figure f3-epih-44-e2022055:**
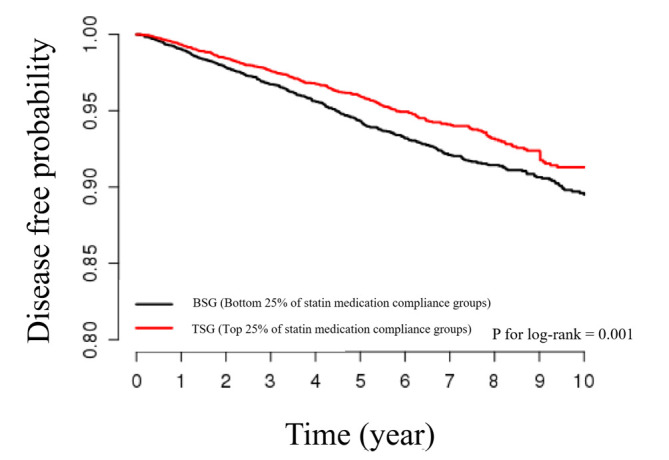


**Table 1.** Baseline characteristics of the study population

Characteristics	Chronic periodontitis patients		p-value
	BSG (n=6,172)	TSG (n=6,172)	
Demographics			
Age (yr)	72.2±5.5	72.2±5.1	0.47
Sex (male)	2,018 (31.7)	2,018 (31.7)	1.00
Income level (quartile)			0.55
First	1,354 (21.9)	1,393 (22.6)	
Second	880 (14.3)	878 (14.2)	
Third	1,476 (23.9)	1,412 (22.9)	
Fourth	2,462 (39.9)	2,489 (40.3)	
Underlying disease			
Hypertension	5,325 (86.3)	5,297 (85.8)	0.48
Diabetes	1,930 (31.3)	1,982 (32.1)	0.32
Charlson comorbidity index	4.3±3.0	4.3±2.8	0.18
Statin medication			
Disease to medication interval (day)^1^	90.3±94.8	90.8±96.8	0.78
Medication possession ratio	0.3±0.2	1.0±0.0	<0.001
Outcome			
Ischemic stroke	408 (6.6)	279 (4.5)	<0.001

Values are presented as mean±standard deviation or number (%). BSG, bottom 25% of statin compliance group; TSG, top 25% of statin compliance group.

1 The interval between the date that chronic periodontitis was diagnosed and the date that a statin was prescribed.

**Table 2.** Associations between statin use and the incidence of ischemic stroke

Variables	Events	Follow-up duration (person-years)	Incidence rate (per 1,000 person-years)	Crude	p-value	Adjusted^1^	p-value
All (n=12,344)					0.001		0.002
BSG	408	35,560	11.47	1.00 (reference)		1.00 (reference)	
TSG	279	31,350	8.90	0.78 (0.67, 0.91)		0.79 (0.67, 0.92)	
Male (n=4,036)					0.170		0.240
BSG	159	11,811	13.46	1.00 (reference)		1.00 (reference)	
TSG	121	10,517	11.51	0.85 (0.67, 1.07)		0.87 (0.68, 1.10)	
Female (n=8,308)					0.002		0.002
BSG	249	23,749	10.48	1.00 (reference)		1.00 (reference)	
TSG	158	20,833	7.58	0.73 (0.60, 0.89)		0.73 (0.60, 0.90)	
Age ≥85 (n=7,788)					0.020		0.010
BSG	263	25,445	10.34	1.00 (reference)		1.00 (reference)	
TSG	186	22,738	8.18	0.79 (0.66, 0.96)		0.78 (0.65, 0.94)	
75≤ Age <85 (n=4,301)					0.060		0.090
BSG	135	9,538	14.15	1.00 (reference)		1.00 (reference)	
TSG	89	8,296	10.73	0.77 (0.59, 1.01)		0.79 (0.61, 1.04)	
65≤ Age <75 (n=255)					0.560		0.580
BSG	10	577	17.33	1.00 (reference)		1.00 (reference)	
TSG	4	316	12.66	0.71 (0.22, 2.26)		0.72 (0.22, 2.32)	

Values are presented as hazard ratio (95% confidence interval). BSG, bottom 25% of statin compliance group; TSG, top 25% of statin compliance group.

1 Adjusted for age, sex, income level, hypertension, diabetes, and Charlson comorbidity index.

## SUPPLEMENTARY MATERIALS

Supplementary Material 1. Definitions of diseases

Supplementary Material 2.

Supplementary Material 3. Subgroup analysis of associations between statin use and the incidence of ischemic stroke
