# Supplementary material for: The risk of stroke according to statin medication compliance in older people with chronic periodontitis: an analysis using the Korea National Health Insurance Service-Senior Cohort Database
Source: Epidemiol Health. 2022 Jul 5;44:e2022055. doi: 10.4178/epih.e2022055 (PMC9754917; doi:10.4178/epih.e2022055)
Supplement: Supplementary Material 1. — Definitions of diseases [file epih-44-e2022055-suppl1.docx]

**Supplementary Material 1. Definitions of diseases**

| **Disease** | **ICD-10 codes** | **Claim codes** |
| --- | --- | --- |
| Chronic periodontitis | K053 | Dental procedures including U2232, U2233, U2240, U1010, U4411, U4412, U1051, U1052, U1072, U1072, U1081, U1082, U1083 |
| Hypertension | I10, I11 | ≥1 prescription of anti-hypertensive drugs ≥ 1 |
| Diabetes | E10, E11, E12, E13, E14 | ≥1 prescription of anti-diabetic drugs ≥ 1 |
| Ischemic stroke | I63, I64 | ≥1 admission |

ICD-10, International Classification of Diseases, 10th revision.
