# Supplementary figures and images for: The risk of stroke according to statin medication compliance in older people with chronic periodontitis: an analysis using the Korea National Health Insurance Service-Senior Cohort Database
Source: Epidemiol Health. 2022 Jul 5;44:e2022055. doi: 10.4178/epih.e2022055 (PMC9754917; doi:10.4178/epih.e2022055)

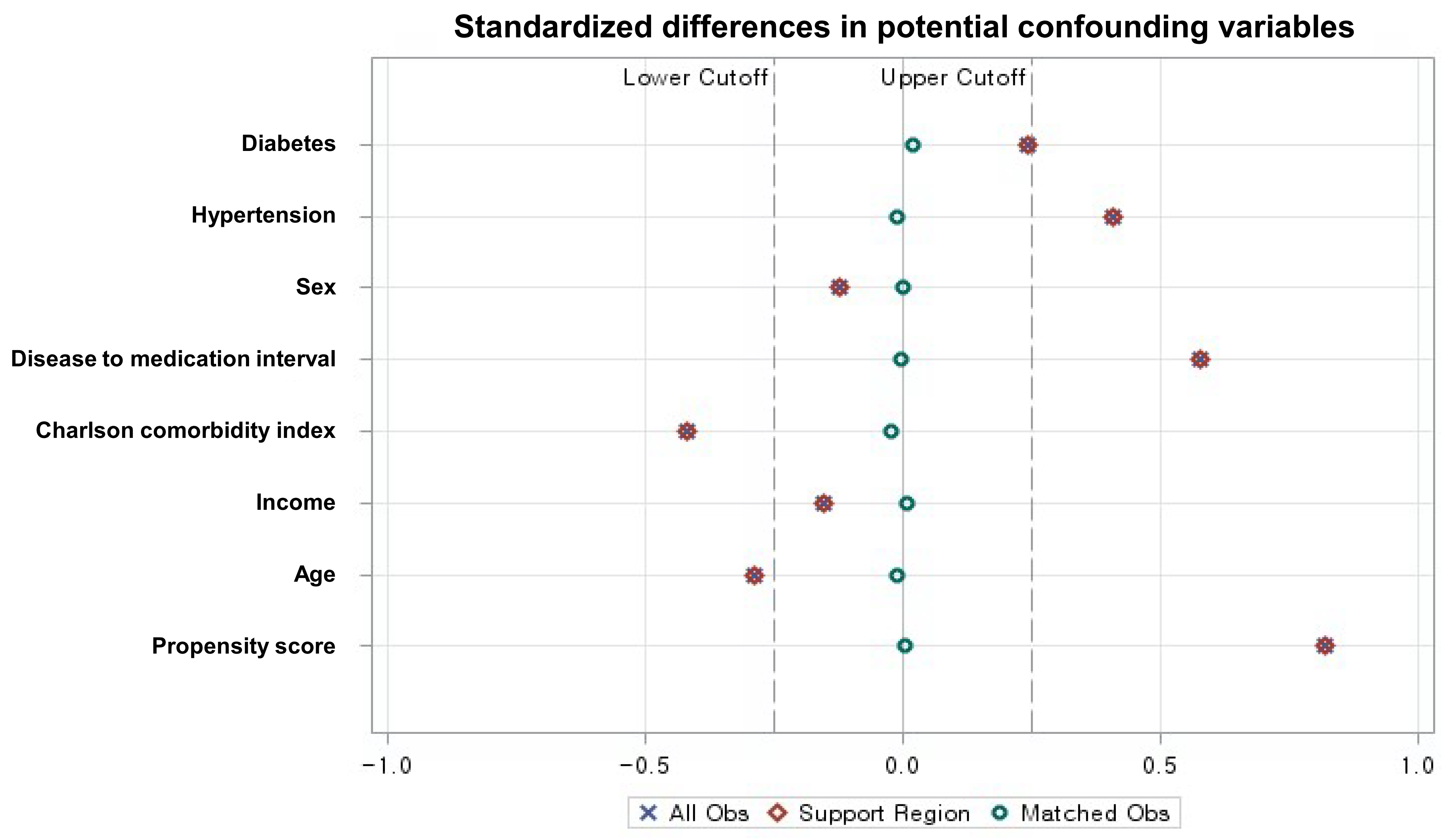

Supplement: Supplementary Material 2. [file epih-44-e2022055-suppl2.tif]
