# Supplementary material for: The risk of stroke according to statin medication compliance in older people with chronic periodontitis: an analysis using the Korea National Health Insurance Service-Senior Cohort Database
Source: Epidemiol Health. 2022 Jul 5;44:e2022055. doi: 10.4178/epih.e2022055 (PMC9754917; doi:10.4178/epih.e2022055)
Supplement: Supplementary Material 3. — Subgroup analysis of associations between statin use and the incidence of ischemic stroke [file epih-44-e2022055-suppl3.docx]

**Supplementary Material 3. Subgroup analysis of associations between statin use and the incidence of ischemic stroke**

|  | **Events** | **Follow-up duration (person-years)** | **Incidence rate (per 1000 person-years)** | **Hazard ratio (95% confidence intervals)** | | | |
| --- | --- | --- | --- | --- | --- | --- | --- |
|  |  |  |  | **Crude** | ***P* value** | **Adjusted*** | ***P* value** |
| **All**  **(n = 12,344)** |  |  |  |  |  |  |  |
| BSG | 408 | 35560 | 11.47 | 1.00  (reference) |  | 1.00  (reference) |  |
| TSG | 279 | 31350 | 8.90 | 0.78  (0.67-0.91) | 0.001 | 0.79  (0.67-0.92) | 0.002 |
| **Hypertension**  **(n = 10,622)** |  |  |  |  |  |  |  |
| BSG | 381 | 30496 | 12.49 | 1.00  (reference) |  | 1.00  (reference) |  |
| TSG | 252 | 26825 | 9.39 | 0.76  (0.64-0.89) | < 0.001 | 0.76  (0.65-0.90) | < 0.001 |
| **No hypertension**  **(n = 1,722)** |  |  |  |  |  |  |  |
| BSG | 27 | 5064 | 5.33 | 1.00  (reference) |  | 1.00  (reference) |  |
| TSG | 27 | 4525 | 5.97 | 1.13  (0.66-1.94) | 0.64 | 1.10  (0.64-1.89) | 0.73 |
| **Diabetes**  **(n = 3,912)** |  |  |  |  |  |  |  |
| BSG | 167 | 10822 | 15.43 | 1.00  (reference) |  | 1.00  (reference) |  |
| TSG | 107 | 10017 | 10.68 | 0.70  (0.55-0.89) | 0.004 | 0.73  (0.57-0.94) | 0.01 |
| **No diabetes**  **(n = 8,432)** |  |  |  |  |  |  |  |
| BSG | 241 | 24738 | 9.74 | 1.00  (reference) |  | 1.00  (reference) |  |
| TSG | 172 | 21333 | 8.06 | 0.83  (0.69-1.01) | 0.07 | 0.82  (0.67-1.00) | 0.05 |

*Adjusted for age, sex, income level, hypertension, diabetes, and Charlson comorbidity index.

BSG, bottom 25% of statin compliance group; TSG, top 25% of statin compliance group.
